# Realising the potential of participatory research in youth mental health: Time to go back to basics

**DOI:** 10.1002/jcv2.12292

**Published:** 2024-11-23

**Authors:** Lucy Biddle, Zoë Haime

**Affiliations:** ^1^ Department of Population Health Sciences University of Bristol Bristol UK; ^2^ The National Institute for Health Research Applied Research Collaboration West (NIHR ARC West) at University Hospitals Bristol and Weston NHS Foundation Trust Bristol UK; ^3^ Biomedical Research Centre University Hospitals Bristol and Weston NHS Foundation Trust Bristol UK

**Keywords:** co‐creation, involvement, mental health, participatory research, young people

## Abstract

The importance of embedding participatory methods within youth mental health research is well accepted and often a funding prerequisite. However, we argue that there is a need to revisit the core values of the approach in order to ensure that participatory methods remain meaningful, effective and authentic. This should entail rigorously examining ‘why’ and ‘how’ to approach participatory methods ‐ not merely outlining the ‘what’ and ‘when’. We need to prioritise the naturalistic epistemic advantage youth can bring to our research, assigning them a unique collaborative role to reflect this rather than seeking to professionalise our participants. Similarly, we should explore innovative methods to empower youth to apply their lived experience and knowledge alongside the researcher. Equally, we must continue to reflect on our roles as researcher, ensuring we develop the skills necessary for participatory research with children and young people.

**Key points**

- Participatory methods are commonly used and expected in youth mental health research.
- Challenges exist regarding a lack of common guidance for effective youth involvement in research.
- We argue there is a need to revisit core values underlying participatory research to make this fully authentic and beneficial.
- Participatory method evaluations in studies with children and young people are underreported. Critical reflection of our current practices is essential to progress this field and identify innovative methods for involvement.

Few would challenge the importance of embedding participatory methods within our research designs, particularly as we attempt to represent the voices and respond to the needs of some of the most vulnerable children and young people (CYP) in our societies. Indeed, nowadays youth participation or co‐production is typically a funding prerequisite and tokenistic involvement no longer cuts the mustard. We might even suggest that academics newly approaching the field may become overwhelmed by the burgeoning literature, but what are we actually learning from one another and how do we make our attempts at participatory research truly authentic?

As well as showcasing examples of research co‐produced with young people, the authors in this special issue grapple with several challenges. These include flagging a lack of common understanding or guidance on best approaches for planning and conducting participatory research (Jones et al., 2024), particularly when working with marginalised or vulnerable groups of young people, such as those with neurodiversity or who are primary school‐aged (Bartnick et al., 2024). Some attempt to derive their own co‐produced protocols (Bartnick et al., 2024), while others advocate for further research. Questions are similarly raised around reporting practices, transparency and replicability (Bakermaus‐Kraneburg & van IJzendoorn, 2024). In Bakermaus‐Kraneburg and van IJzendoorn's review, only 25% of studies met the criteria for replicability which, it is argued, may lead to premature translation of co‐produced research findings. Others highlight a need to establish how to foster a supportive and respectful culture for credible youth participation and to consider how young people can benefit from their participatory role (Jones et al., 2024).

These studies then, all touch on the necessity of ensuring participatory methods are meaningful and effective, but how do we ensure our research is meeting these requirements? First, we must start by revisiting the core values of the approach. This means recognising that the theoretical basis for participatory involvement of children and young people in research is grounded in the foundations of empowerment, democratic principles, and social change.

Most would agree that balancing power dynamics should be the basis of the approach, moving beyond researching ‘on’ to researching ‘with’ participants, yet challenges remain in consistently creating environments where all feel valued and empowered to contribute. This is evident in funding arrangements where researchers are encouraged to use participatory methods to develop ideas and research questions before securing grant money, often relying on participant goodwill and charity and so immediately making their role inequitable (Mittelmark, 2007). These expectations may also assume participants have sufficient knowledge of research processes to contribute effectively from the outset, which places an onus on participants to adapt or learn quickly, reinforcing an imbalance where the researcher's experience is privileged (Hanley et al., 2003). Additionally, short turnaround times for funding calls, including those encouraging participatory approaches, overlook the significant time required to plan meaningful activities and build authentic relationships within teams including participatory researchers ‐ especially where broad age ranges exist (Cook, 2012).

Further, researchers have complicated the definition of what constitutes participatory involvement. Some argue that true participation should entail equal engagement in all aspects of the research process, from conception to conclusion, following a rights‐based approach (Alderson, 2008). Arguably, this perspective aligns with article 12 of the United Nations Convention on the Rights of the Child (UNCRC), which asserts that CYP have the right to actively participate in all decisions that affect them, by expressing their views, and being heard (Lundy & McEvoy, 2009). Others have acknowledged that this attempt to balance the equilibrium is not a realistic expectation, particularly when considering engagement and involvement across all age groups (Lundy et al., 2011).

Instead, to genuinely democratise the participation of CYP in research, we should be prioritising their unique lived experience contributions and perspectives, rather than their research skills. Crucially, our youth in participatory research can bring epistemic advantage (Carabello et al., 2017). As such, they have the power to unlock methodological aspirations by embedding the ultimate form of ‘*verstehen’* within our research—a deep form of empathetic understanding described by Sociologist, Weber, that emerges from walking in another's shoes and which was traditionally sought by sending the researcher out into the field to themselves participate in the world that they researched. Youth participation can ‘bring the field into research’, creating for us a way to deliver robust, targeted and responsive research that is arguably more ethical than participant observation, is authentic (Davidson, 2017), and which avoids the threat and compromise of the investigator ceasing to function as a researcher and instead ‘going native’.

However, we will not access this by disproportionately recruiting individuals with future aspirations for higher education, or those who have become ‘professional participants’ (Fisher, 2019). Relying on these groups risks the creation of an echo chamber of similar perspectives and experiences across CYP research. Limiting the diversity of voices means we may also inadvertently marginalise those with unique perspectives, which could include CYP from diverse socioeconomic, health, or educational backgrounds. Instead, we must work to seek innovative ways to promote inclusivity and harness the full spectrum of lived experiences within our collaborative teams (Rix et al., 2010). Similarly, we will not benefit fully from youth inclusion without identifying a suitable role and method of involvement for our participants that empowers them to apply their lived experience and knowledge alongside the researcher. Once again, we must strip back to basics, asking ourselves *how* we will conduct participatory research during the course of our projects.

We must avoid removing the naturalistic quality of involvement work by professionalising our involved youth, assigning tasks which expect them to mimic (with minimal training) the actions of researchers. We should also challenge the comfortable formula of involvement workshops or steering groups. However, the question of ‘how’ brings more challenges as ‐ although frameworks for action exist and continue to be developed ‐ there remains a lack of consensus on the most effective and appropriate methods to use in CYP participatory designs (Freire et al., 2022). Recently, we have undertaken creative methods as part of a participatory approach to our Digital Dialogues project, which seeks to facilitate communication between mental health professionals and their young patients about the online world and online activity (www.digitaldialogues.co.uk). These innovative activities facilitated discussion and stimulated valuable content, which our CYP with lived experiences of mental health issues were able to use as they went on to conceptualise and create knowledge exchange resources to inform mental health practitioners. However, such methods need to be undertaken with caution. There is a risk that creative methods, alike to other approaches, can quickly steer away from true participatory involvement by limiting engagement in shaping the research direction and providing only superficial opportunities to consult on work (Sevón et al., 2023). This can quickly turn participatory involvement into ‘stakeholder’, ‘end‐user’ or ‘advisor’ roles, instead of the ‘co‐creator’ or ‘co‐researcher’ roles, which give them authentic ownership over project ideas, control, and outcomes.

To ensure we are providing a genuine participatory experience it is then also important to be transparent and clear about *why* we chose our method, and to critically evaluate not only project outcomes but also participants' experiences in the collaborative role (Bradbury‐Jones et al., 2018). Given that participatory method evaluations in studies with CYP are underreported, we must encourage critical reflection of our current practices as a field, and share insights where possible (Montreuil et al., 2021). This approach will help us develop sustainable and effective solutions for conducting participatory research, whilst also continuously reflecting on our role as researchers, making replication and meaningful changes to methodological decisions viable.

In conclusion, while we may now instinctively accept the received wisdom that youth participation is a good idea and should be integrated into our studies, to do this properly and to do this justice, we must go back to the fundamentals, reassess and critique. We must rigorously ask *why* and *how* we will approach participatory methods ‐ not merely outline the *what* and *when.* This requires recognising that the young people we involve, like other expert members on our multidisciplinary team, bring a unique contribution and so need to be assigned an appropriately unique collaborative role befitting of this. Equally, it is important that we continue to reflect on our role as researcher, ensuring we have or can develop the skills necessary for participatory research with CYP.

## AUTHOR CONTRIBUTIONS

**Lucy Biddle:** Conceptualization; writing ‐ original draft; writing ‐ review and editing. **Zoë Haime:** Conceptualization; writing ‐ original draft; writing ‐ review and editing.

## CONFLICT OF INTEREST STATEMENT

The authors have declared that they have no competing or potential conflicts of interest.

## ETHICAL CONSIDERATIONS

None.

## Data Availability

Data sharing not applicable to this article as no datasets were generated or analysed.
